# Case of Compound Fracture at the Ankle-Joint

**Published:** 1823-11

**Authors:** Robert Brown

**Affiliations:** Member of the Royal College of Surgeons, &c. London.


Art. IV.—*Case of Compound Fracture at the Ankle-Joint.* By Robert Brown, Esq. Member of the Royal College of Surgeons, &c. London.

Surgery, as well as the other departments of medicine, has undergone many important improvements, even within the present century: the cultivation of anatomy, the basis of scientific medicine, and the persevering attention to pathology, have led to the possession of important principles in the treatment of diseases.

When men of liberal education and extended observation unite together for its cultivation, it is but reasonable to expect that advancement should be made in this branch of practical knowledge: it is, however, much to be lamented that the number is so few of those who are truly ardent in the pursuit, and zealous for the attainment of that preliminary requisite Anatomy, which alone can qualify them for the successful practice of medicine and surgery. The time, I trust, is not far distant when society will perceive the necessity of rescuing the practice of surgery from those whose early life has been occupied in pursuits inimical to fitting them for the profession of a surgeon. Whilst men of dishonest principles and consummate ignorance engage in the treatment of cases requiring extensive acquaintance with the structure and economy of the parts subject to disease, numerous errors must continually be committed, and lives often sacrificed, by such audacious meddlers; tampering thus "with diseases of which they know little, and with bodies of which they know less." I am borne out by daily occurring facts, which warrant these assertions, and forcibly demonstrate the necessity of legislative interference to prevent the commission of further mischief. Manifest are the improvements that have taken place since the regulations adopted by the Apothecaries' Society; and I doubt not an equally beneficial consequence would be found to result from the adoption of similar regulations by the College of Surgeons. From the testimonials of qualification which this respectable body requires from candidates for a diploma, it is expected that a good foundation for that knowledge shall have been laid, which future practice and observation will enable them to acquire. Thus fitted for extensive general information, with a laudable ardour for the profession, no one will surely presume to assert that they are not better prepared for professional employment than those less acquainted with the grand principles of surgical science. Thus a want of restrictions on those who practise surgery gives tacit sanction to the presumptuous and arrogant to impose, by specious promises and false pretences, on the credulous and unwary valetudinarian. Pott very justly remarks, that "the desire of health and ease, like that of wealth, seems to put all understandings and all men upon a level: the avaricious are duped by every bubble, the lame and the unhealthy by every quack. Each party resigns his understanding, swallows greedily, and for a time believes implicitly the most groundless, ill-founded, and delusory promises; and nothing but loss and disappointment ever produces conviction."

In no one subject, as I think, has surgery received more decided improvement than the diseases, and particularly the injuries, to which the bones and joints are obnoxious; most of their diseases have been so minutely, and their treatment so well ascertained; and in injuries, the circumstances which warrant us to attempt preservation with the most appropriate mode of practice, have been so ably and so correctly laid down, that that mutilation and loss of limbs, which a few years ago was so common, is now comparatively unfrequent.

Anxious to promote the interests of surgical science, I request, with your permission, the insertion of the following history; the successful termination of which will add one more to the number in testimony of the progressive improvement of modern surgery: and I am further induced to lay the case before the public, because it has come to my knowledge that the practice which I pursued on this occasion was condemned, by some who were of opinion that I ought immediately to have removed the limb, rather than have attempted its preservation.

Ann Coulthurst, ætatis 10, in riding upon a wooden horse of a circular machine, September 10th, 1822, became sick from its revolving motion, and was thrown from her seat with considerable violence to the ground; immediately after which, it is said she was carefully conveyed home. On my arrival, about six o'clock P.M., I found a compound fracture of the left tibia near its extremity, connected with the astragulus, and about four inches of bone projecting through the lacerated integuments; the fibula also, as might be expected, was transversely fractured about three inches from the joint, but unconnected with any wound of the soft parts. Both malleoli were loose, from laceration of their principal ligamentous attachments. The hemorrhage was but trifling, and apparently venous, with great coldness of the foot. Mr. Liddell, a professional gentleman on a visit here, and who accidentally followed the crowd that had assembled around her dwelling, to inquire into the circumstances of the accident, most kindly offered me his assistance. After due inquiry into the several circumstances of the case, as all things seemed favourable, we determined to attempt the preservation of the limb: we therefore began the operation of reducing the protruded bone, which, after some difficulty, by gently fleeting back the leg, we effected without the necessity of enlarging the size of the wound.

The patient was then put to bed, and a pillow placed under the limb, upon which was laid a pasteboard splint, softened and moulded to the shape of the leg; a linseed-meal cataplasm, made with beer-grounds, was then laid upon the injured part, and the leg kept moderately secure with the many-tailed bandage; over these again it was deemed expedient to place a deal splint, which extended on the fore-part of the limb, from the patella to the instep. An opiate, with tinct. opii, m. xx. was given; and the same quantity was repeated about ten P. M.

September 11th.—Has passed a tolerably good night; pulse 114; slight hemorrhage since last night; a firm coagulum fills up the wound. Cataplasm renewed.—℞. Pulver. G. Scammon., Hydrargyri Submur., Pulv. Cinnamon. C. sing. gr. v. Fiat pulvis, statim sumendus ex quovis idoneo vehiculo. Gave strict charge to confine the patient to cool re-gimen, and to exclude all unnecessary and officious visitors.

Vespere.—Cataplasm renewed. Has been disturbed twice by the operation of the powder. Tinct. opii, m. xl. to be taken at bed-time.

12th.—Night has been spent in a restless way; wound looks well; little tumefaction of the limb; heat but little increased. Constitutional symptoms are, however, greater than yesterday; pulse 130; bowels confined.—℞. Magnesiæ Sulphatis, ʒx.; Infusi Sennæ, ℥v.; Aquæ Menthæ Pip. ℥j. ft. Mistura cujus sumantur cochlearia duo magna pro re nata, ad alvum solvendam. Cataplasm renewed; and the following lotion directed to be constantly applied to the limb:—℞. Liq. Plumbi Subacetatis, ℥j.; Aceti Distillati, ℥ijss.; Aq. Pluvialis, ℈ij. Fiat lotio.

Vespere.—Pulse 140; bowels not moved since yesterday; skin moist, tongue covered with a white coat. Cataplasm renewed. Opiate to be repeated at bed-time.

13th, 11 A.M.—I was this morning accompanied by my friend Dr. Moore, who continued to the end of the case with most diligent attention, and anxiously participated with myself for the well-doing of the patient, whose welfare only we had now to consider; for, conceiving, as we had good right to do, that our professional character was unusually concerned by the ungenerous insinuations of a contemporary practitioner, who, on more occasions than this, has evinced the same feeling, spared neither his influence nor exertions to prejudice the friends, and shake their confidence in our competency to the proper management of the case.—Patient this morning very irritable and cross; pulse 160; bowels moved several times since last visit; instep very much tumefied; from the undue pressure of the wooden splint, which is directed to be discontinued; wound looks well. Cataplasm and lotion to be continued, and the aperient medicine to be administered as occasion may require.

Vespere.—A little more tension of the foot; constitutional symptoms augmented. Cataplasm renewed.

14th.—Pulse very rapid; tongue more moist at the edges; tension of the limb is greater, extends upwards, and is accompanied with a general erythematous inflammation, which has proceeded to vesication on the outside of the leg. Admoveantur hirudines, xvi. Aperient repeated and cataplasm renewed, and the poppy fomentation directed to be substituted for the lotion.

Vespere.—Much easier with regard to the limb; is very fretful and refractory.—℞. Tincturæ Opii, m. xxxv.; Liquoris Antimonii Tartar. m. xx.; Mist. Camphoræ, ℥j. fiat haustus. Hora somni sumendus.—Cataplasm renewed.

15th.—Has had a very comfortable night's rest; the limb somewhat easier; a great number of vesicles have formed, with yellowness around their bases; the erythematous blush somewhat greater; the large vesicle that was noticed yesterday has burst; the wound little altered in appearance; pulse 140, tongue improved. Aperient repeated, also the cataplasm and fomentation.

Vespere.—Inflammation of the limb more extensive, and the lymphatics on the interior part of the thigh inflamed and tense, and the inguinal glands connected with them enlarged and painful. The vesicles noticed in the morning increased to a very large size, and numerous smaller ones appear on the inflamed part of the leg. The serum in the vesicles seems partially coagulated. The larger bullæ punctured; sixteen more leeches have been applied, after which the limb became less tense. The dressings renewed; anodyne to be repeated.

16th.—Night has been spent very comfortably; pulse 140; general appearance of the patient better; tension of the limb greatly abated; the first-formed bullæ pours out bloody pus. Dressings, &c. renewed.

Vespere.—Has rested well all day, and seems much improved.

17th.—Pulse 120; tongue changed for the better; bowels open; the large vesicle continues to discharge. The vesication stopped; and the tension and inflammation of the limb nearly subsided. The wound at the ankle beginning to discharge pus. Dressings renewed.

Vespere.—The day has been spent free from pain; symptomatic fever nearly gone; the discharge of good pus increased more in quantity since the morning; the coagulum filling the wound is separating. Dressings as before.

18th.—Much improved in every respect. Dressings renewed twice.

19th.—Wound looks exceedingly well, and discharges laudable pus. A contusion on the outer part of the leg, immediately below the fractured fibula, has ulcerated; partly, probably, in consequence of the original injury, and partly also from the weight of the limb on this already injured part. Tension over the instep entirely gone, and the integuments very much corrugated. Pulse 118; tongue granulated and clean; bowels open. Cataplasm renewed.

Vespere.—All things seem to be going on favourably. Dressings, &c. renewed.

20th.—Night has been passed very comfortably; bowels open; pulse 128. In every respect appears much better.

21st.—Is still improving.

22d.—She has had a good night. A slight increase of inflammation in the neighbourhood of the wound. Bowels constipated: a dose of the aperient mixture directed to be given.

23d.—The state of the limb is not so favourable as it was a few days past; the inflammation is somewhat augmented, and on the inferior and outer part of the limb an ulcer of some depth, whence pus is freely discharged, presented itself on the removal of the cataplasm. Pulse 130; bowels acted on twice during the night; skin soft, tongue moist. Dressings as before.

Vespere.—The limb has undergone no perceptible change since morning. Bowels open; fæces of a pitchy colour and offensive odour. Laxative to be repeated.

24th.—Has had a good night; the inflammatory condition of the limb is much diminished; discharge from the wound of a thicker consistence. Pulse 112; bowels have a tendency to become constipated.—℞. Hydrargyri Submuriatis, gr. iv.; Pulveris Antimonialis, gr. ij.; Confectionis Sennæ, q. s. fiat bolus. Sumatur cum cyath. mist. purg. olim præsc. Dressings renewed.

Vespere.—Bowels have been moved once. Bolus and mixture to be repeated.

25th.—In every respect doing well: bowels have, however, a tendency to become constipated, and require frequent use of laxative medicines. Dressings as before.

26th.—Much as she was yesterday.

28th.—Bowels have been freely opened; pulse reduced to 90; wound discharges copiously. Ulcer on the outer malleolus enlarging. Dressings renewed.

30th.—Bowels comfortably opened; very prominent granulations fill up the wound at the inner ankle, which seems to increase. Dressings renewed.—℞. Decocti Cinchonæ, ℥vj.; Extracti Glycyrrhizæ, ʒij. solve et adde Acidi Sulphurici diluti, m. xx. fiat M. Capiat cochl. amplum pro dosi 4tâ. q. 9 horâ.

October 1st.—Pulse for some days past has been about 90. Bowels open. A small tumor, seemingly containing a fluid, has begun to form over the inner ankle. Wound dressed with lint, and the cataplasms employed cold.

4th.—Nothing particularly worthy of notice has taken place for the last few days, except a slight cough on the 2d instant, for which she took at bedtime the following draught:—℞. Tincturæ Opii, m. xx.; Liquor. Antimon. Inf. m. xv.; Mist. Camphoræ, f. ℥j.; Syr. Tolutani, f. ʒij. fiat haustus.

5th.—Continues to improve; the tumor on the inner ankle has subsided, having formed a connexion with the primitive wound, which is looking well; granulations greatly reduced since the applications of the lint. Bowels kept open by a three-grain calomel pill, at bedtime. Repetatur mist. cinchonæ comp.

7th.—Exuberant granulations sprinkled with hydrargyri nitrico oxydum. Has been very pettish and irascible for the last two days, and complains of great and frequent twitchings of the limb. Bowels open; discharge from the wound greatly diminished, and the limb possesses greater firmness. Anodyne draught to be repeated at bedtime.

8th.—The cataplasm discontinued; the wounds covered with dry lint, and retained by the many-tailed bandage. General health of the patient very much improved. The decoct. cinch. c., and the aperient, when necessary, to be continued.

9th.—The flabbiness of the limb, from the continued use of the cataplasm and fomentations, much lessened; wounds look well. Soap-plaster applied to the limb morning and evening.

15th.—The ulcer on the external side discharges more freely; a large portion of cuticle has separated, exposing an extensive suppurating surface, which was dressed with simple cerate; the wound on the inner side greatly improved; the exuberant granulations reduced to a level with the surrounding parts. Dressings and the medicines as before.

16th.—The tumor over the inner malleolus has again become tense and painful, and, from a small ulcer in the middle, a small quantity of sero-purulent fluid is discharged. The cataplasm again resorted to.

26th.—Nothing particularly worthy notice has occurred till to-day, when, on laying bare the limb, considerable inflammation was found to occupy the lower part of the leg and foot. Bowels open; wounds look well. Eight leeches directed to be applied, and afterwards the poppy-fomentations. Purgative repeated.

27th.—Night has been spent in a restless way. Complains of great pain in the foot, which is still much inflamed. Thirteen more leeches to be applied.

Vespere.—The inflammation still great. Leech-bites continue to bleed, and are further promoted by the repeated applications of warm fomentations. Pulse exceedingly quick; skin hot and dry; bowels have been three times moved in the course of the afternoon. Head and hands directed to be frequently sponged with cold water. Anodyne to be repeated at bedtime.

28th.—Last night has been spent with much restlessness. The inflammation of the foot little abated. Pulse very quick; bowels moved; skin very hot. Sixteen leeches to be applied.

Vespere.—The inflamed condition of the foot still continuing, twelve more leeches were applied to the instep, and four to the upper part of the leg.—℞. Pulvis Antimonialis, gr. iv.; Hydrarg. Submuriat. gr. ij.; Conf. Rosæ Canin. q. s. fiat bolus. Horâ somni sumendus.

29th.—Has had a bad night. Vomited the bolus as soon as taken. The inflammation, however, seems very much abated. One dejection since last visit. Cataplasm and fomentations to be continued as before.

31st.—The morbid heat of the limb greatly lessened; tongue covered with a white mucous coat; bowels stated to be open. Directed a larger dose of the purgative mixture.

November 1st.—Bowels quite open; limb free from any inflammation.—℞. Decocti Cinchonæ Lancif. ℥vss.; Acidi Muriatici, m. xx. fiat M. Capiat cochl. amplum ter in die.

3d.—In every respect considerably amended; wounds almost healed up. Cataplasms and fomentations still continued; medicines as before.

6th.—For the last two days the cataplasms and fomentations have been discontinued, and dressings of ceratum saponis substituted: there is little or no discharge from the wound on the inner, and the ulcer on the outer malleolus is quite healed up. The decoct. cinchonæ comp. and an aperient to be occasionally continued.

10th.—Has sat up the major part of the day. The discharge from the wound trifling. The simple roller used instead of the many-tailed bandage.

It would be trespassing too much upon your goodness, and the patience of your readers, to continue the daily reports of this case, as the injury is reduced to a simple ulcer. Suffice that I briefly state, that, from the time of the last report, the limb continued gradually to acquire strength, but remained somewhat thicker than the other; and the patient, at the expiration of ten weeks from the receipt of the accident, was able to walk about the house without the aid of any kind of support. A small ulcer over the inner ankle remaining, Baynton's treatment was adopted: the spouting of cold water from a kettle elevated several feet was practised for about a week, when the ulcer healed up; and, at about the end of six months, a very small spicula of bone came away, since which, to the present time, the limb has been equally as strong, and the mobility of the joint as perfect, as the other; and I may say, too, nearly as shapely.

*Preston, Lancashire; September 18th,* 1823.

